# The relationship of sleep complaints risk factors with sleep phase, quality, and quantity in Japanese workers

**DOI:** 10.1007/s41105-017-0110-1

**Published:** 2017-07-21

**Authors:** Yuuki Matsumoto, Naohisa Uchimura, Tetsuya Ishida, Yoshitaka Morimatsu, Mihoko Mori, Miyako Inoue, Nanae Kushino, Michiko Hoshiko, Tatsuya Ishitake

**Affiliations:** 1 Department of Environmental Medicine, Kurume University School of Medicine, 67 Asahi-machi, Kurume, 830-0011 Japan; 2 Department of Neuropsychiatry, Kurume University School of Medicine, Kurume, Japan

**Keywords:** Sleep complaints, Lifestyle, Sleep environment

## Abstract

Numerous studies have determined that lifestyle factors (smoking, drinking, snacking, etc.) and the bedroom environment can influence sleep. We developed a new sleep scale—the 3-Dimensional Sleep Scale (3DSS)—which measures three elements of sleep: phase, quality, and quantity. The purpose of this study is to determine which risk factors of sleep complaints are associated with these sleep elements. Data were obtained from 366 Japanese day workers (302 men and 64 women). Sleep condition was assessed with the 3DSS, and we also assessed various habits within 2 h of going to bed, including smoking, drinking, snacking, caffeine intake, mobile phone use, and working. We also asked about bedroom environmental conditions (noise, lighting, and temperature and humidity). Multivariate logistic regression analysis using the backward selection method (likelihood ratio) was used, with 3DSS scores as the outcome (i.e., over or under the cutoff). The results showed that smoking was associated with significantly greater odds ratio [2.71 (1.65–4.44)] of disordered sleep phase, while lighting as well as temperature and humidity led to greater odds [3.67 (1.55–8.68), 1.93 (1.20–3.11)] of poor sleep quality. Finally, only noise was significantly related to greater odds [1.98 (1.13–3.46)] of low sleep quantity. These findings indicated the various risk factors of sleep complaints could be associated with different sleep elements. This might help in the effective treatment of sleep complaints.

## Introduction

Japanese people are well-known to be hard workers, and they often think that working at the cost of sleep is a virtue. Ironically, sleep is essential for working efficiently and for the health of workers. Obesity, hypertension, diabetes, and depression, all of which are common diseases among workers, are associated with sleep complaints [1–4]. Beyond such health problems, sleep complaints are frequently implicated in workplace problems, such as low productivity, risk of accidents, and conflict in personal relationships [5–8]. Thus, neglect of sleep can lead to considerable disadvantages in both individuals and organizations.

Although individuals with serious sleep disorder must be referred to a medical institution, self-management is required for the majority of cases. Indeed, the major controllable risk factors of sleep disorder are behaviors or activities before bed and the bedroom environment. Smoking, drinking, snacking, and caffeine, which are together referred to as *shiko*-*hin* in Japan, have been implicated in sleep disorders [9–12]. Other relevant behaviors include use of a mobile phone and working before going to bed, which can enhance wakefulness [13, 14]. In terms of environmental risks, there are three major factors—noise, lighting, and temperature and humidity—that must be considered when attempting to achieve good sleep [15–18].

In the past, we established that sleep comprises three measurable elements—phase, quality, and quantity—which we then integrated into an original scale called the 3-Dimensional Sleep Scale (3DSS) [19, 20]. In our current 24-h society, people often exhibit problems in their sleep phase (e.g., regularity and chronotype), as well as in their sleep quality (e.g., sleep efficiency and satisfaction) and quantity (e.g., sleep duration and fullness). Previous studies have reported that sleep phase problems may be related to certain mental disorders independent of sleep quality and quantity [21]. In addition, sleep quality and quantity might have somewhat different relationships with depression [22–24]. In particular, the behaviors related to poor sleep quality (e.g., difficulty falling asleep) and poor sleep quantity (e.g., lack of sleep) are rather different. Given the differing properties of these sleep elements, it would be exceedingly difficult to provide organizational or clinical support correctly if the elements are not considered separately. Therefore, it is important that these sleep elements be assessed separately to fully grasp the details of individuals’ sleep problems. Problematically, most previous studies investigating the influence of bedtime habits or environment have examined only one or two of these three sleep elements (e.g., insomnia, sleep duration, or circadian rhythms); at present, we do not believe that any studies have investigated the specific risk factors of all three sleep elements. The purpose of the present study is to examine which factors are known to be associated with sleep complaints—in particular bedroom habits and environmental factors—more strongly influence these three sleep elements.

## Methods

### Participants

Data were collected in April 2015, with the cooperation of a company (that mainly provided manufacturing services) in Japan. We gave participants the questionnaires at their workplace and then collected the questionnaires later that day. A total of 446 Japanese employees responded; however, we ultimately excluded shift workers and respondents with missing data (*n* = 80), leaving 366 day workers (302 males and 64 females) for analysis. The mean ± standard deviation of age was 43.9 ± 8.8 years (males: 43.8 ± 8.9, females: 44.3 ± 8.3).

### Measures

Sleep condition was measured with the 3DSS. This scale was designed for use with Japanese day workers and its reliability and validity have been established [19]. The 3DSS comprises three categories (sleep phase, quality, and quantity), each comprising 5 items (for a total of 15); all items ask about usual sleep habits in the past month. The sleep phase items are as follows: “(1) I go to bed at a fixed, regular time on weekdays and weekends”; “(2) I wake up at a fixed, regular time on weekdays and weekends”; “(3) I have a well-balanced breakfast every day;” “(4) ‘Morningness’ is better suited to me than is ‘eveningness’”; and “(5) What time do you wake up on weekdays?” The items assessing sleep quality are as follows: “(6) It takes me more than 30 min to fall asleep”; “(7) I wake up more than twice a night”; “(8) I wake up earlier than usual (over 2 h) and cannot fall asleep again;” “(9) I don’t sleep soundly”; and “(10) I worry that I cannot fall asleep”. Finally, the sleep quantity items are as follows: “(11) I sleep for less than 6 h on weekdays”; “(12) I cannot get enough sleep even though I want to”; “(13) I don’t feel free from sleepiness or fatigue when I wake up”; “(14) I feel sleepy not only in the afternoon, but also in the morning and/or evening;” and “(15) I often doze off”. To answer the items, respondents must choose the response option that best fits their sleep habits from among four choices, as follows (except for item no. 5): (1) always, (2) often, (3) rarely, and (4) never. For item no. 5, the options are (1) about 6:00 a.m. or earlier than 6:00 a.m., (2) about 6:30 a.m., (3) about 7:00 a.m., and (4) later than 7:00 a.m. As for the scoring, answers (1)–(4) are given scores of 3–0, respectively, for the sleep phase items (no. 1–5), and scores of 0–3 for the sleep quality and quantity items (no. 6–15). Each category’s score ranges from 0 to 15, with higher scores indicating better sleep condition. The cutoff scores for disordered sleep were 8/9 for sleep phase and quantity and 10/11 for sleep quality [25].

Bedtime habits were assessed as follows: “Please check all behaviors that you usually perform within the 2 h before going to bed: smoking, drinking, snacking, intake of caffeine, mobile phone use, and working”. For bedtime environmental factors, we assessed noise, lighting, and temperature and humidity in the bedroom. The specific questions and response options are the follows: “How noisy is your bedroom when you go to bed?” (1 = silent; 2 = noisy, but it does not prevent me from sleeping; 3 = noisy and it prevents me from sleeping). “How is the lighting in your bedroom when you go to bed?” (1 = dark; 2 = lit, but it does not prevent me from sleeping; 3 = lit and it prevents me from sleeping). “How is the temperature and humidity in your bedroom when you go to bed?” (1 = comfortable; 2 = uncomfortable, but it does not prevent me from sleeping; 3 = uncomfortable and it prevents me from sleeping). Only two participants selected option 3 for the noise question, and no one selected 3 for the other two questions. Thus, we decided to classify participants into two groups: those who selected 1 [i.e., environmental risk (−)] and those who selected 2 or 3 [environmental risk (+)].

### Statistical analyses

IBM SPSS Statistics 23 was used for the analysis, with a significance level set at *p* < 0.05. We used unpaired *t* tests and analyses of variance (ANOVAs) as univariate analyses. Then, a multivariate logistic regression was run, using the backward selection method (likelihood ratio), to calculate odds ratios (ORs) and 95% confidence internals (CIs).

### Ethical considerations

We ensured that none of the participants were pressured or harmed due to nonparticipation, and made it known that their responses to the questionnaires constituted informed consent to participate. The subjects received no reward for participation. Personal data were strictly monitored to ensure confidentiality and protect participants’ privacy. This study was approved by the Kurume University Ethics Review Board and informed consent was obtained.

## Results

Table 1 shows the 3DSS scores according to gender, age, and marital status, as well as the number and percentage of participants whose 3DSS scores were under the cutoff points. A significant difference between men and women was found for sleep quality score only, with women showing a higher score than men. There were significant differences in all 3DSS scores according to participants’ age. Specifically, sleep phase score increased with age. Multiple comparisons revealed significant differences between participants in the over-50 group and participants in all other ages (*p* < 0.001); we also found a significant difference between participants aged under 29 and those aged 40–49 (*p* = 0.004). Significant differences were also apparent between the 30–39 and over-50 (*p* = 0.003) groups in sleep quality score and between the 40–49 and over-50 (*p* = 0.022) groups in sleep quantity score. We observed significant differences between single and married participants in sleep phase scores.

**Table 1:** Mean ± standard deviation (SD) of 3DSS scores by gender, age, and marital status

	*n*	Sleep phase	Sleep quality	Sleep quantity
Total	366	9.5 ± 3.2	10.3 ± 3.2	7.9 ± 2.9
Under cutoff point; *n* (%)		135 (36.9)	177 (48.4)	209 (57.1)
Gender				
Men	302	9.4 ± 3.2	10.1 ± 3.2	8.0 ± 2.8
Women	64	9.6 ± 3.2	11.3 ± 2.8	7.2 ± 3.3
*p*		0.630	0.009*	0.036*
Age				
Under 29	27	7.3 ± 3.2	10.7 ± 2.6	8.1 ± 3.5
30–39	70	8.3 ± 3.2	11.3 ± 2.8	7.6 ± 2.5
40–49	173	9.4 ± 3.1	10.2 ± 3.3	7.6 ± 2.9
Over 50	96	11.0 ± 2.7	9.6 ± 3.1	8.6 ± 2.8
*p*		<0.001^†^	0.005^†^	0.030^†^
Marital status				
Married	254	9.8 ± 3.0	10.4 ± 3.2	8.0 ± 2.8
Single	94	8.3 ± 3.3	10.1 ± 3.2	7.5 ± 3.1
Other	11	10.6 ± 3.5	11.7 ± 3.1	8.2 ± 3.5
*p*		<0.001^†^	0.259	0.394

* Significant differences by unpaired *t* test

^†^Significant differences by ANOVA test

Table 2 shows the 3DSS scores according to bedtime habits. Sleep phase score was lower among those who were smoking and who used a mobile phone, while sleep quality score was higher among those who used a mobile phone. Sleep quantity scores were lower in those who did not drink, those who used a mobile phone, and those who worked.

**Table 2:** Mean ± standard deviation (SD) of 3DSS scores by bedtime habits

	*n*	Sleep phase	Sleep quality	Sleep quantity
Drinking				
(−)	253	9.5 ± 3.1	10.5 ± 3.1	7.6 ± 2.9
(+)	113	9.4 ± 3.5	10.0 ± 3.2	8.5 ± 2.7
*p*		0.889	0.139	0.010*
Smoking				
(−)	249	9.9 ± 3.0	10.4 ± 3.2	7.9 ± 2.9
(+)	117	8.4 ± 3.2	10.1 ± 3.1	8.0 ± 2.9
*p*		<0.001*	0.345	0.734
Snacking				
(−)	294	9.5 ± 3.2	10.4 ± 3.2	7.9 ± 2.9
(+)	72	9.2 ± 3.2	10.1 ± 3.0	7.9 ± 2.8
*p*		0.445	0.491	0.969
Caffeine intake				
(−)	318	9.6 ± 3.2	10.3 ± 3.2	7.9 ± 3.0
(+)	48	8.7 ± 3.2	10.7 ± 2.9	7.7 ± 2.4
*p*		0.059	0.410	0.459
Mobile phone use				
(−)	161	10.0 ± 3.0	9.9 ± 3.4	8.5 ± 2.9
(+)	205	9.0 ± 3.2	10.7 ± 3.0	7.4 ± 2.8
*p*		0.001*	0.019*	<0.001*
Working				
(−)	352	9.5 ± 3.2	10.3 ± 3.2	8.0 ± 2.9
(+)	14	9.6 ± 3.4	10.8 ± 2.9	5.7 ± 2.3
*p*		0.885	0.571	0.004*

* Significant differences by unpaired *t* test

Table 3 shows the 3DSS scores according to bedroom environment. Sleep quality scores significantly differed by all environmental factors, while only noise showed a significant difference in sleep quantity score.

**Table 3:** Mean ± standard deviation of 3DSS scores by bedroom environment conditions

	*n*	Sleep phase	Sleep quality	Sleep quantity
Noise				
(−)	290	9.6 ± 3.2	10.5 ± 3.1	8.1 ± 2.9
(+)	75	9.0 ± 3.2	9.6 ± 3.4	7.2 ± 2.9
*p*		0.153	0.020*	0.019*
Lighting				
(−)	335	9.5 ± 3.2	10.5 ± 3.1	7.9 ± 2.9
(+)	31	8.7 ± 3.4	8.7 ± 3.4	7.3 ± 2.7
*p*		0.140	0.003*	0.253
Discomfort from temperature and humidity				
(−)	253	9.5 ± 3.2	10.7 ± 3.0	8.0 ± 2.9
(+)	113	9.4 ± 3.1	9.4 ± 3.4	7.6 ± 2.8
*p*		0.860	<0.001*	0.130

* Significant differences by unpaired *t* test

Table 4 shows the results of the multiple regression analysis with 3DSS scores (over or under the cutoff score) set as the outcome. We found that participants who smoked had greater odds of poor sleep phase scores; snacking was also retained in the final step, but it was not significant. Furthermore, participants in the lighting (+) and temperature and humidity (+) groups both had greater odds of poor sleep quality. Finally, while mobile phone use, working, and noise remained in the final step of the analysis, only noise (+) had a significantly greater odds of low sleep quantity.

**Table 4:** Odds ratios and 95% confidence intervals for under the cutoff points of 3DSS scores

	Sleep phase		Sleep quality		Sleep quantity	
	OR (95% CI)^a^	*p*	OR (95% CI)^a^	*p*	OR (95% CI)^a^	*p*
Bedtime habits						
Drinking						
(−)						
(+)						
Smoking						
(−)	1.00					
(+)	2.71 (1.65–4.44)	<0.001*				
Snacking						
(−)	1.00					
(+)	1.70 (0.95–3.03)	0.072				
Caffeine intake						
(−)						
(+)						
Mobile phone use						
(−)					1.00	
(+)					1.51 (0.99–2.33)	0.059
Working						
(−)					1.00	
(+)					4.50 (0.98–20.6)	0.053
Bedroom environmental conditions						
Noise						
(−)					1.00	
(+)					1.98 (1.13–3.46)	0.016*
Lighting						
(−)			1.00			
(+)			3.67 (1.55–8.68)	0.003*		
Discomfort from temperature and humidity						
(−)			1.00			
(+)			1.93 (1.20–3.11)	0.007*		

^a^Final step of multivariate logistic regression with the backward selection after adjustment for gender, age, and marital status

* Significant difference (omnibus test, *p* < 0.05; Hosmer–Lemeshow test, *p* > 0.1)

## Discussion

We demonstrated which risk factors of sleep complaints (bedtime habits and environment) are associated with the three elements of sleep (phase, quality, and quantity). Whereas previous sleep studies tended to cover only one or two elements of sleep, we investigated all three and found that they were associated with different risk factors. This suggests that it is necessary to assess sleep phase, quality, and quantity separately to clarify the causes of sleep complaints and respond appropriately. Compared to a previous study [20], the prevalence of poor sleep phase in this study was lower while that of poor sleep quality or quantity were similar. Specifically, the previous study reported that the prevalence of poor sleep phase was about 44%, whereas in this study, it was 37%. The difference might be because the average age of the participants in this study was higher than was that of the previous study, given that younger individuals tend to have lower sleep phase scores.

Smoking was found to be the strongest risk factor of sleep phase problems. Nicotine has an arousal function and a half-life of around 2 h [26], which means that people who smoke within 2 h of going to bed might stay up later. Additionally, changing the number of cigarettes or start time of smoking can result in irregular sleep onset. Snacking was retained in the final step of the regression analysis, and although it was not significant, this result suggests that its connection is stronger than are those of the other risk factors. Snacking before bed might make people feel less hungry in the morning, and thus can cause them to skip breakfast. Skipping breakfast can make it difficult to synchronize the periphery circadian rhythm with the central circadian rhythm [27, 28], thereby leading to irregular sleep patterns and an eveningness lifestyle. Another possibility is that people who were classified as having an eveningness lifestyle tend to like *shiko*-*hin* [29] and pay little attention to their health. Therefore, a lack of health-consciousness might underlie the relationships between poor sleep phase, smoking, and snacking. The above-mentioned can apply to people with irregular sleep based on inadequate sleep hygiene rather than the patients suffering from delayed sleep-phase disorder.

As for sleep quality, lighting was the strongest risk factor, followed by temperature and humidity. The production of melatonin is inhibited even by normal room light (particularly over 100 lx) [30]. During sleep, light that is over 30 lx can impair both slow-wave and rapid-eye movement (REM) sleep [31]. While actual bedroom illuminance could not be measured in this study, it was still found to have a significant impact on the odds of reporting poor sleep quality. Regarding temperature and humidity, unsuitable temperatures (i.e., excessively hot or cold) can make it difficult to stay asleep and impairs both slow-wave and REM sleep [17]; high humidity can also make it difficult to stay asleep and can impair slow-wave sleep [18]. Because skin temperature increases with perspiration and the core body temperature must decrease to sleep soundly, unsuitable temperature and humidity would disturb the functioning of these systems.

Interestingly, most participants who reported these environmental risk factors did not feel that the factors actually prevented them from sleeping. This is perhaps because participants felt that even when the factors were present, they were not a problem if participants had no difficulty in falling asleep. Nevertheless, the impaired slow-wave and REM sleep due to inappropriate lighting or temperature and humidity typically manifest as difficulty in maintaining sleep rather than difficulty in falling asleep.

Noise was the strongest risk factor of sleep quantity. Noise louder than 40 dB during sleep can activate autonomic responses and increase cortisol levels on wake-up, thus leading to excessive daytime fatigue, sleepiness, and declining performance [15]. Additionally, at around 5 h after sleep onset, sleep is typically in the shallow stages (stage 1–2), which means that people are awoken more easily by noise and their sleep duration is thus cut short. Furthermore, these physical responses do not adapt to chronic exposure to noise, whereas subjective sleep satisfaction can [15]. These facts indicate why noise influences sleep quantity to a greater degree than sleep quality.

Importantly, mobile phone use and working remained at the final step of the regression analysis, albeit without significance. Some previous studies have shown that there is a relationship between lack of sleep or tiredness and these practices [13, 14, 32]. This is perhaps because they capture people’s attention before sleep, thus reducing their sleep duration. It is possible that a significant association between sleep quantity and these practices would be found if a larger sample were used. Working, in particular, had the largest effect size of all factors. It is likely that overwork could reduce sleep duration more than any other lifestyle or bedroom environment factor. Furthermore, blue light can reduce production of melatonin and thereby delay sleep phases or lead to declines in sleep quality [33]; however, some specialists have proposed that the amount of light coming from a mobile phone at a moderate distance has rather little effect on melatonin production [34]. Nevertheless, these past studies offer some support as to why mobile phone use was associated with sleep quantity, rather than sleep phase or quality.

The present study shows that the risk factors of sleep complaints appear to be associated with different sleep elements. *Shiko*-*hin*, such as smoking and snacking, is closely related to lifestyle rhythm. Lighting as well as temperature and humidity may be primarily related to the first stages of sleep (i.e., slow-wave sleep appearing), whereas noise is related to the last stages (i.e., shallow sleep and waking up easily). The differences in the characteristics of these factors might underlie their differing relationships with the various sleep elements. Therefore, when identifying patterns of sleep complaints, it would be important to focus on the risk factors present before attempting treatment.

### Limitations

This study has some limitations. First, we could not infer any causal relationships between sleep elements and risk factors because the study was cross-sectional. Second, the data on noise, lighting, and temperature and humidity were not detailed because these factors were not objectively measured with equipment. Finally, we included no information on mental or physical diseases or medications being taken, or other factors important for workers such as educational attainment and job rank. In addition, the number of participants, especially in the under-29 age group and women, was insufficient to reflect the characteristics of the wider population. These are important to consider because sleep architecture and work styles differ between the young and old, as well as between men and women. Further research with more participants using stratified sampling, and including data on diseases and medicines, would be needed to better establish the associations found herein.
